# Violent conflict and breastfeeding: the case of Iraq

**DOI:** 10.1186/s13031-019-0244-7

**Published:** 2019-12-30

**Authors:** Vidya Diwakar, Michael Malcolm, George Naufal

**Affiliations:** 1 Overseas Development Institute, 203 Blackfriars Road, London, SE1 8NJ UK; 2 West Chester University, 700 S High Street, West Chester, PA 19382 USA; 3 Public Policy Research Institute, 4476 Texas A&M University, College Station, TX 77843-4476 USA; 4 Institute of Labor Economics (IZA), Bonn, Germany

**Keywords:** Conflict, Breastfeeding, Middle East, Iraq, D74, I1, J13, J18

## Abstract

**Background:**

This study explores the relationship between armed conflict and breastfeeding practices of Iraqi mothers. To date, the relationship between violent conflict and breastfeeding is surprisingly understudied. Especially in the Middle East, which is conflict-prone and has a young population, research on war and household behavior is critical for promoting recovery and sustainable development.

**Methods:**

This study employs a unique pairing of the Iraq Body Count Database and the 2006 and 2011 Multiple Indicator Cluster Surveys for Iraq. We use probit models to explore the association between armed conflict and several breastfeeding outcomes – whether a child was ever breastfed, whether a child was breastfed within 1 h after birth, whether a child is currently breastfed, and whether an infant under 6 months of age is exclusively breastfed. Our proxies for conflict intensity are the average rate of conflict-related casualties across the 3 years prior to survey administration and the rate of casualties averaged across the 2 years prior to the birth of the child, in the governorate in which the family resides. We employ a number of other independent variables important for breastfeeding status, including health controls and characteristics of the household, child and mother. We also use a Cox proportional hazards model to study the association between conflict and breastfeeding duration. We complement this analysis with various robustness checks, including disaggregation by year, controls for household wealth and an analysis of breastmilk substitutes and their potential for an interaction with household wealth.

**Results:**

We find in our main results that increases in conflict-related casualties are associated with a significant decline in the probability that a child was ever breastfed and a decline in the probability that a child is currently breastfeeding. There is no significant association with exclusive breastfeeding or with initiation of breastfeeding within 1 h after birth. This result is robust to alternative measures of conflict, although some coefficients from estimation based on the 2006 subsample are positive and not significant, and reverse causation is a potential source of bias in interpreting cross-sectional feeding patterns. Results on breastfeeding duration are mixed. Our results also suggest an increase in the use of breastfeeding substitutes like formula concurrent to higher levels of conflict among wealthier households.

**Conclusion:**

The results are informative in the context of designing policy aimed at stabilizing the long-term health and productivity of populations in conflict areas. Infant formula provided with the objective of offering temporary relief creates risks, including reducing the probability and duration of breastfeeding. Attention to the supply of health care and to support systems for women, especially skilled breastfeeding support and targeted support to infants dependent on formula, are matters of the utmost urgency during and after conflict periods.

## Introduction

The impact of war on the physical and psychological health of affected populations is long-lasting and severe, and unfortunately young children often bear the brunt of these costs [60]. In this paper, we use data from Iraq to explore the relationship between armed conflict and breastfeeding, which is an important but understudied aspect of the impact of war on children.

Most literature supports the contention that breastfeeding is important for child health. Black, et al. [12], using data from a number of low-and middle-income countries, estimate that optimal breastfeeding has the potential to prevent over 800,000 deaths annually in children under the age of five. Children who are exclusively breastfed are on average 14 times less likely to die in the first 6 months after birth, with substantially lower risk of contracting diseases like diarrhea and acute respiratory infections [11]. Breastfeeding also confers immunological protection and reduces the risk of chronic conditions later in life, including obesity, high cholesterol and diabetes [12, 47]. For women who are able to breastfeed, potential disadvantages of deviating from the practice appear to go beyond child health risks. Del Bono and Rabe [18] and Fitzsimons and Vera-Hernandez [29], exploiting exogenous variation in the availability of breastfeeding support, find that breastfeeding is associated with a number of improvements in both cognitive and non-cognitive indicators later in childhood. In summary, disruptions to breastfeeding have the potential to reduce human capital and productivity, and thus standard of living, over the long run.

Turning to the health effects of war, it is well-known that violent conflict leads to a host of detrimental effects on civilian populations living in combat zones. These consequences are especially dire for children, and there is a growing empirical literature on the subject. Davis and Kuritsky [17] find, using data from Sub-Saharan Africa, a decline in life expectancy of about 1 year in countries that experienced a conflict killing at least 1000 people, while Plumper and Neumayer [48] argue that women suffer from war more than men do. Bell et al. [10] document an increase in anxiety disorders associated with conflict-related violence in Cambodia. Specifically with respect to children, Al-Eissa [6] finds that children in Kuwait displayed dysfunctional social and emotional behaviors that were influenced by their experience of aggression during the Gulf conflict in 1990. Alderman et al. [5] investigate the preschool nutritional status of children in Zimbabwe during the civil war there, finding negative impacts. Akresh et al. [2] find that the civil war in Rwanda was associated with a reduction in girls’ height-for-age. Similarly, Bundervoet et al. [13] find that children born in Burundi in areas with high conflict intensity are likely to experience a height-for-age deficiency compared to those in non-conflict areas. Akresh et al. [4] further document this finding with evidence from the Eritrean-Ethiopian conflict, in which war-exposed children were found to be shorter. Mansour and Rees [41] use Palestinian data to observe that conflict-related deaths are associated with an increase in the probability of having a baby with low birthweight, subsequently impacting the long-term health of these children. Kesternich et al. [37] argue that war experience is a predictor of both economic and health outcomes at older ages. While these studies document the detrimental effects of conflict on child outcomes, less is known about the transmission mechanisms and about the impact of war on micro-level, child-specific health indicators like breastfeeding [17].

Overall, there is a substantial body of literature on the benefits associated with breastfeeding, and also a growing literature that studies the negative health consequences associated with war, particularly among children. However, there is almost nothing that analyzes the link between the two. Theoretically, the effect of armed conflict on breastfeeding is ambiguous. On one hand, high stress and poor nutrition can lead to a decline in breastfeeding. On the other hand, disruptions to the food supply chain and to the health infrastructure might spur an increase in breastfeeding out of a lack of other options.

From our reading of the literature, Zakanj et al. [66], Summers and Bilukha [54], and Guerrero-Serdan [32] are the only studies that touch on the relationship between war and breastfeeding. Zakanj et al., using descriptive statistics from survey data collected in Croatia, find that conflict is associated with a reduction in both the prevalence and duration of breastfeeding, and argue that this is most likely due to the distribution of infant food by international organizations. Summers and Bilukha [54], using a survey of internally displaced persons in the Ukraine, find that conflict-related stress is the most frequently reported reason for early breastfeeding termination. Guerrero-Serdan [32] briefly explores the effect of the 2003 Iraq war on breastfeeding, only insofar as it acts as a transmission channel to poor nutritional outcomes of children, which is the focus of that paper.

Iraq is a prime location to study this association. Armed conflict has been a dominant feature of Iraq’s contemporary history. A succession of three major wars since the 1980s, harsh sanctions and internal political strife have led to extensive damage to the country’s infrastructure. Conflict has reduced access to healthcare services and facilities, led to the deterioration of the quality of drinking water and sewage systems, and disrupted the provision of food and other basic necessities [32]; in 2003 alone, 12% of Iraq’s hospitals were damaged [22]. Damage to the electricity infrastructure has only compounded the disruption to health services [8]. Iraq also has a particularly young population. In 2015, the median age of an Iraqi was 19.3 years, which places it among the 40 youngest countries in the world and at the top of the Middle East and North Africa (MENA) region, along with Palestine and Yemen [61]. Richards and Waterbury [52] estimate that the number of excess deaths of Iraqi children under the age of five between 1991 and 2002 was somewhere between 345,000 and 529,000, which they attribute mostly to direct and indirect consequences of the sanctions regime. The authors do mention inadequate breastfeeding as a potential contributory factor underlying those deaths.

The MENA region is a particularly interesting context in which to study breastfeeding, as it features one of the lowest exclusive breastfeeding rates in the world [34], and is the only region where breastfeeding rates fell in the decade leading up to 2006. While 39% of infants under 6 months old are exclusively breastfed in the developing world overall, the figure is 26% in the MENA region [57]. Al-Nuaimi et al. [7] provide evidence of continuing declines until 2012 in some MENA countries. This is despite the fact that the Quran (2:233) explicitly counsels breastfeeding for two full years. Various reasons have been proposed for this apparent discrepancy. Radwan [51] has cited the influence of marketing of formula in the region. Teshome and Takea [56] note the extensive distribution of infant formula in Iraq by aid organizations, which was ongoing from 1996 to 2017 and has been linked to infant malnutrition (Emergency Nutrition [24]), along with inadequate funding for breastfeeding support. Growing labor force participation among women (Galtry [30]) and a lack of women’s empowerment to make these decisions autonomously may also contribute. We ask in this paper whether regional conflict can help to explain low breastfeeding rates.

Exploring this link makes an important contribution. The MENA region is conflict-prone and has a bulging youth population [21].^1^ Existing literature suggests that children experience drastic consequences from conflict, many of which have high persistence into adulthood. Studying the mechanisms that underlie these effects, potentially including disrupted breastfeeding, is therefore a crucial health policy question.

## Methods

### Participants, setting, and data

The secondary household data are drawn from the Multiple Indicator Cluster Surveys (MICS), developed by the United Nations Children’s Fund, which aim to capture information on the status of women and children in various countries around the world. In Iraq, the surveys are conducted via face-to-face interview by the Central Organization for Statistics & Information Technology and Kurdistan Regional Statistics Office, in collaboration with the Ministry of Health. The sampling is stratified over 56 geographical sample domains to ensure coverage of rural and urban areas in all governorates, along with all major metropolitan areas. Within each sample domain, primary sampling units (essentially neighborhoods) are selected at random, and a cluster of six households is selected at random from each neighborhood. The sample is designed to be roughly nationally representative, per census data.

In this study, we use secondary, publicly available data from the 2006 and 2011 rounds of the survey, which comprise the data available following the 2003 US invasion. 17,873 households were sampled in 2006 and 35,701 households in 2011. The response rates are quite high – 98.6% for the 2006 survey and 99.6% for the 2011 survey. Ultimately, our dataset consists of information on children under the age of two. There are 2088 such children in the 2006 sample and 13,071 in the 2011 sample. The random sampling of households is conducted across physical domicile structures at the time of the surveys, rather than by census registries of individuals, so by definition physically displaced persons are not recorded at their original homes. We discuss the implications in section 4. The 2000 wave of the Iraq MICS survey is also publicly available, but we do not have any governorate-specific data on conflict prior to 2003 that are comparable to the data we use in this paper.

### Variables

#### Outcome variables – breastfeeding status

Our primary dependent variables take advantage of several questions from the MICS survey instrument, asked of all mothers who gave birth in the 2 years prior to the survey, about their last-born child. For each child in the sample, we construct the following binary outcome variables:

1. Was the child ever breastfed?
2. Was the child breastfed within 1 h after birth?
3. Is the child exclusively breastfed? (no solids or liquids other than breastmilk in the previous 24 h)
4. Is the child currently breastfed?

These outcome variables are motivated by World Health Organization guidelines, which recommend that breastfeeding be initiated within 1 h after birth, and that infants 6 months of age and younger be exclusively breastfed. Breastfeeding should then continue at least until the age of two, possibly together with other foods [59]. To reflect these recommendations, we restrict our analysis of question (3) to infants under 6 months of age. We also disaggregate our analysis of question (4) into one subsample including only infants less than 6 months old and to a second subsample including children aged between 6 months and 24 months.

We also directly examine whether the child consumes breastmilk substitutes (BMS) by constructing a binary variable equal to 1 if the child consumed milk or infant formula in the day preceding the survey. We restrict this analysis to infants less than 6 months old, to focus on the period for which exclusive breastfeeding is recommended.

#### Independent variables

Our primary independent variable of interest is a household-specific measure of armed conflict intensity. To measure conflict, we merge each individual household record with casualty numbers by governorate from the Iraq Body Count (IBC) database. IBC provides statistics and timestamps on deaths that have occurred across governorates since the onset of war in 2003. The data are mainly based on media reports, but also on primary sources, nongovernmental organizations and official figures.

We employ two measures of conflict intensity. The average casualty rate measures casualties per 1000 population, averaged across the 3 years preceding the MICS survey, in the governorate in which the respondent lives. The pre-birth casualty rate measures casualties per 1000 population, averaged across the 2 years prior to the birth of the child, in the governorate in which the respondent lives.

We also employ a number of additional controls for breastfeeding practices, including health-related controls (binary variables reflecting low birthweight, caesarean delivery, antenatal care with skilled health personnel, childbirth with skilled health personnel and contraception use^2^), characteristics of the mother (age, age difference between husband and wife,^3^ total number of children, number of children who passed away and binary variables reflecting primaparous birth, child marriage, primary education, secondary education and employment status), child characteristics (gender), household characteristics (binaries reflecting female-headed household and whether the household is in an urban area) and dummies for whether the household is located in a Kurdish, Sunni or Shia governorate. Table 1 contains a summary of all dependent and independent variables used in our study.

**Table 1:** Description of variables

Variable	Description
Conflict	
Average casualty rate	Average conflict-related casualty rate, per 1000 population, in three years preceding survey, in governorate of household
Pre-birth casualty rate	Average conflict-related casualty rate, per 1000 population, two years prior to birth of child, in governorate of household
Breastfeeding	
Child ever breastfed	Binary indicating if child has ever been breastfed
Hours to breastfeeding	Binary indicating if the child was breastfed within one hour of birth
Exclusive breastfeeding	Binary indicating if the child is only breastfed (=1) or receives any other liquids or solids (=0)
Current breastfeeding	Binary indicating if child was breastfed at time of survey
Milk or infant formula	Binary indicating if child is receiving milk or infant formula
Health	
Low birthweight below 2.5 kg	Binary indicating if child had low birthweight at birth, defined as being below 2.5 kg
ANC with skilled personnel	Binary indicating if mother received antenatal care by a health professional (doctor, midwife, or nurse) or licensed birth attendant
Childbirth with skilled personnel	Binary indicating if child was delivered by a health professional or licensed birth attendant
Birth by caesarean section	Binary indicating if mother had a caesarean section
Contraception	Binary indicating if mother or father is currently practicing one of the following methods of contraception: sterilization, pill, IUD, injections, implants, condom, diaphragm, foam/jelly, lactational amenorrhea method, periodic abstinence, withdrawal
Individual/household	
Male child	Binary indicating if the child is male
Children ever born	Number of children ever born to the mother
Dead children	Number of children born to mother who passed away
Primiparous mother	Binary indicating if the child is a firstborn
Child marriage	Binary indicating of the mother was married before 18 years
Husband-wife age difference	Age difference between husband and wife in years
Age of mother	Age of mother in years
Primary education of mother	Binary indicating if mother completed primary education
Secondary education of mother	Binary indicating if mother completed secondary education
Mother works	Binary indicating if the mother is employed
Female household head	Binary indicating if the household head is female
Regional	
Urban	Binary indicating if household resides in an urban (=1) or rural (=0) area
Kurd	Binary indicating if household resides in a Kurd-dominant governorate, found in north-east Iraq
Sunni	Binary indicating if household resides in a Sunni-dominant governorate, found in the middle and north-west Iraq

### Empirical design: primary interventions

#### Breastfeeding status – Probit regression model

We employ the probit regression model to study the binary outcome variables of interest that we described in section 2.2.1.

$$ \Pr \left({Breastfeed}_i=1\right)=\Phi \left({\beta}_0+{\beta}_1{Conflict}_i+{\beta}_2{H}_i+{\beta}_3{I}_i+{\beta}_4{R}_i+{u}_i\right) $$

Here, *Breastfeed*_*i*_ is the respective outcome variable reflecting breastfeeding status of child *i*. *Conflict*_*i*_ is the rate of conflict-related casualties in the governorate in which the child resides. *H*_*i*_, *I*_*i*_ and *R*_*i*_ are vectors of health, individual/household and regional controls, respectively.

Throughout our estimations, we use robust standard errors, clustered at the level of the governorate. We regard a coefficient to be significant if its corresponding *p*-value is *p* < 0.05 and to be borderline significant if 0.05 < *p* < 0.1. To aid in the interpretation of the estimated coefficients, we also report marginal effects for each independent variable, which reflect the increase in the probability that the outcome variable is equal to 1 associated with a one-unit increase in the respective independent variable, evaluated at the mean values of the independent variables. All statistical analysis was conducted using Stata 14.

The empirical strategy in estimating these models is to exploit locational and temporal variation in conflict levels across surveyed households to identify the association between conflict intensity and breastfeeding practices. This is not an ideal experimental design where households are placed at random in areas with different levels of conflict, and there remains a risk that families in different governorates are endowed with unobserved characteristics that are correlated both with conflict levels and with breastfeeding practices. As such, while we are not able to say that *β*_1_ reflects the true causal impact of conflict on breastfeeding practices, a whole host of control variables, along with a number of robustness checks, help us to measure the association in as robust a way as possible given the limitations of the dataset.

#### Breastfeeding duration – cox proportional hazards model

In addition to breastfeeding status, we are also interested in its duration. While the survey does not offer a retrospective picture of breastfeeding duration for each woman surveyed, we can study breastfeeding duration in an indirect way by using information on the child’s age and whether the child is currently breastfed. To do so, we employ a Cox proportional hazards model. Here, the risk of breastfeeding termination (hazard) in month *t* is given by:

$$ h(t)={h}_0(t)\cdotp \exp \left[{\beta}_1{Conflict}_i+{\beta}_2{H}_i+{\beta}_3{I}_i+{\beta}_4{R}_i+{u}_i\right] $$

The vector of independent variables is defined in the same way as in the probit regressions, and we adopt the same conventions with respect to significance levels. Here, to aid in the interpretation of the coefficients, we report the hazard ratio for each independent variable, which reflects the relative increase in the probability of breastfeeding termination in a given month associated with a one-unit increase in the respective variable. For example, a hazard ratio of 1.2 would reflect a 20 percentage point (ppt) increase in the risk of breastfeeding termination in any given month associated with a one-unit increase in the respective independent variable.

### Robustness tests: alternative interventions

#### Residual analysis by governorate

As an initial check on the robustness of the correlation between conflict and breastfeeding status, we exploit our two survey waves to examine the relationship between *changes* in breastfeeding and *changes* in the level of conflict-related casualties across governorates. To do this, we record the residual for each observation in each of our regressions and then average these residuals over all observations in each governorate, for both years. We also record the average casualty rate within the governorate for both years. We then plot, for each governorate, the change in the mean residual across years against the change in the casualty rate across years. We repeat this analysis for each regression. Among other things, these scatterplots illustrate whether the association in the data between breastfeeding changes and changes in conflict holds across governorates or whether it is driven by a few outliers.

#### Alternative measures of conflict

To further check the robustness of our results, we consider two alternative measures of conflict, as described in 2.2.2. Our main results measure conflict using the average of casualty rates in the 3 years prior to survey administration. We also consider the average casualty rate in the 2 years prior to the child’s birth. The purpose of this alternative measure is to provide variation in conflict levels even within survey years, owing to children of different ages included in the sample.

#### Disaggregation by year

Because of potential differences in the setting, we show results disaggregated by year, estimating our models using the 2006 and 2011 survey waves separately.

#### Wealth controls

In the specifications above, a conspicuously absent covariate is a direct measure of the household’s socioeconomic status, which is potentially an important predictor of breastfeeding [35, 40]. While unfortunately no household wealth index was collected in the 2006 survey, the 2011 survey includes a household wealth indicator, by quintile. Thus, we run our main regressions again, using the 2011 survey wave, but also including controls for household wealth. The indicator for the highest wealth quintile is omitted.

#### Use of breastmilk substitutes

The main analysis studies various indicators of breastfeeding practices, but we also directly examine the provision of breastmilk substitutes (formula or animal milk) for infants under the age of 6 months as a complementary analysis. For these regressions, we include interaction terms between casualty rates and wealth quintiles to determine whether the association between conflict and BMS is potentially intermediated by the household’s wealth level. As in our other analysis that incorporates household wealth, we omit the indicator for the highest wealth quintile.

## Results

### Descriptive statistics

**Fig. 1:**
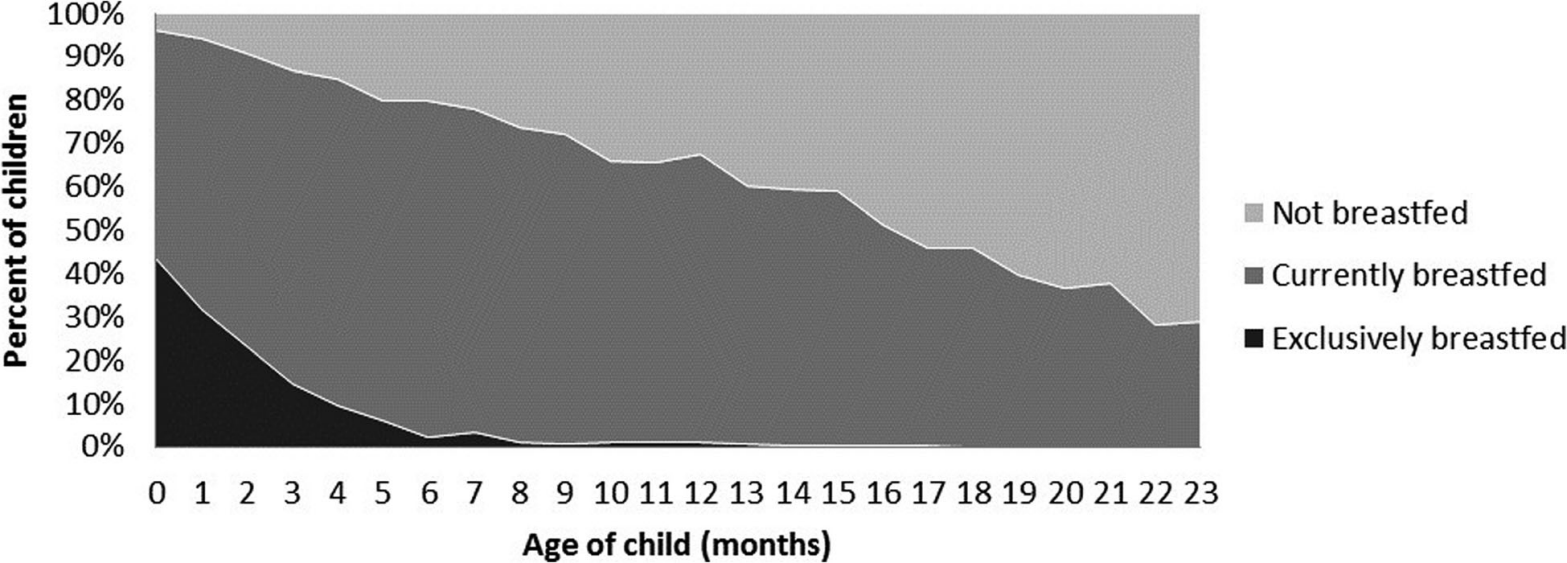
Breastfeeding practices by age (pooled 2006 and 2011 sample)

Figure 1 presents a preliminary descriptive analysis of breastfeeding practices by child age, using pooled data from our 2006 and 2011 samples. Exclusive breastfeeding is quite uncommon. While just over 40% of infants in their first month are exclusively breastfed, this figure drops precipitously to 6% by the fifth month. Moreover, continued breastfeeding is also at lower levels than advised; by 12 months of age, only 66% of children are breastfed at all, with this rate dropping to 29% by 24 months of age.

**Fig. 2:**
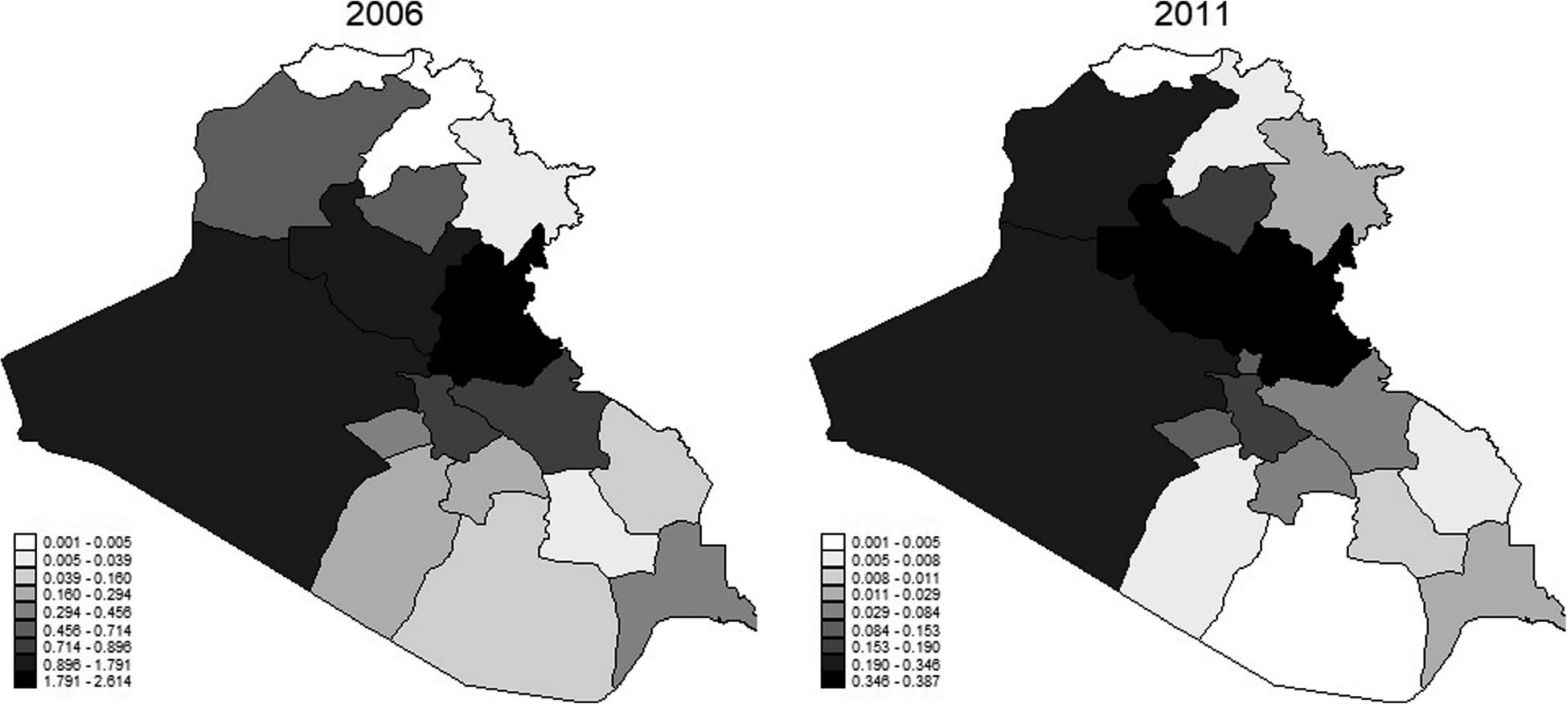
Armed Conflict Casualties in Iraq (per thousand population), 2006 and 2011

**Fig. 3:**
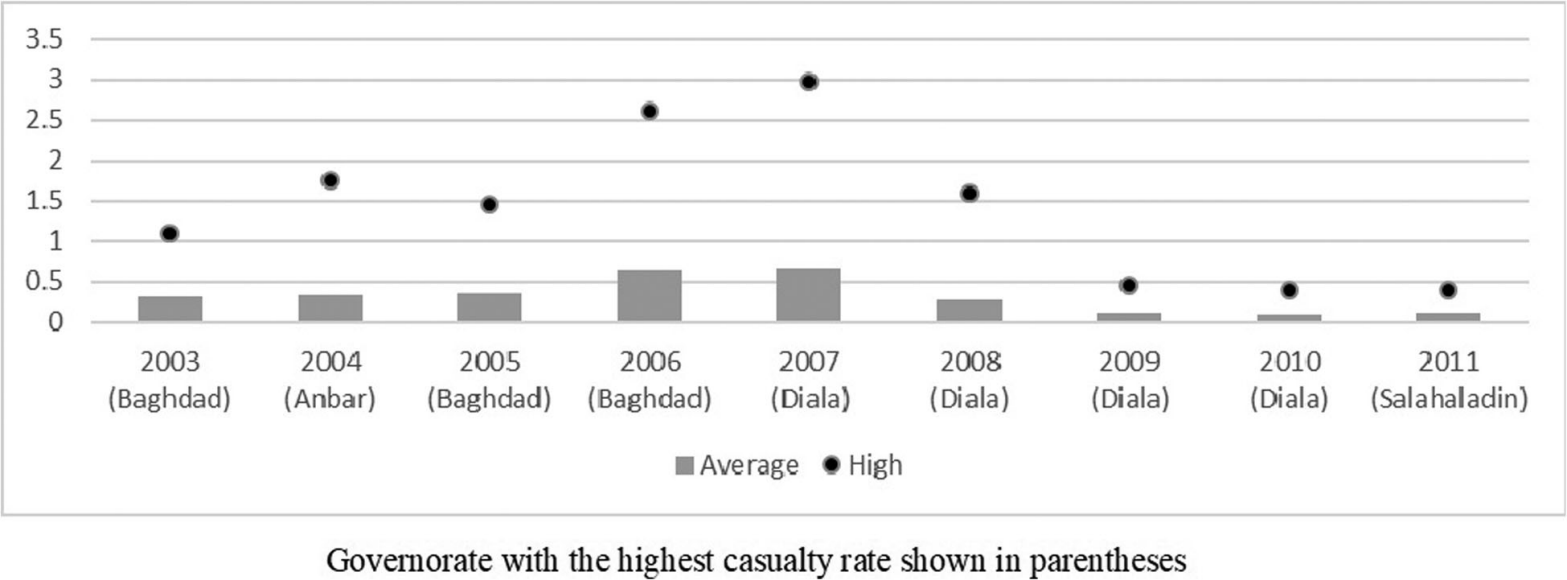
Casualty rate (per thousand population), average and high, 2003–2011

With respect to casualty rates, the mean casualty rate across our sample is around 0.20 casualties per 1000 population. For purposes of our analysis, it is important to note that conflict intensity was not homogeneously distributed across Iraq’s 18 governorates, nor across years. Iraq consists of three main groups: Kurdish, Sunni and Shia. Each group is heavily present in adjacent governorates, practically dividing the country into three distinct sections. The Kurds dominate the northeast part, Sunnis constitute a majority in the center and northwest, while Shia are dominant in the south. Figure 2 shows the intensity of conflict across Iraq for 2006 and 2011, where darker colors indicate a higher rate of war-related casualties. It is clear from this figure that Kurdish and Shia areas were relatively safer than the Sunni-dominated region. For a picture of temporal variation, Fig. 3 displays the average casualty rate per 1000 population for each year between 2003 and 2011, as well as the rate for the governorate listing the highest casualty rate for that year. The highest rate of casualties was in Diala in 2007, with almost 3 war-related casualties per 1000 people. Note also that the geographical distribution of conflict intensity was not homogeneous over the years we are studying. In a period of just 9 years, four different governorates featured the highest annual rate of casualties among the 18 governorates. While there was a notable decline in casualties in later years, there remains a wide range of conflict-affectedness across governorates. For example, there were 0.39 casualties per 1000 population in Sunni-majority Salahaladin in 2011, but only 1 casualty *total* in the Shia-majority Al Muthana governorate and 5 casualties in the Kurdish-dominated Dohuk governorate.

Table 2 contains descriptive statistics on all dependent and independent variables used in this study. Descriptive statistics are presented for the full sample, and also for the subsamples consisting of infants less than 6 months old and children from 6 to 24 months.

**Table 2:** Summary statistics

	Entire sample		Under 6 months		6 months- 2 years	
Variable	N	Mean	N	Mean	N	Mean
Conflict						
Average casualty rate	15,142	0.20	4497	0.21	10,318	0.20
Pre-birth casualty rate	14,815	0.17	4497	0.19	10,318	0.17
Breastfeeding						
Child ever breastfed	15,134	0.93	4496	0.95	10,316	0.93
Breastfed within one hour	13,971	0.44	4236	0.42	9579	0.45
Exclusive breastfeeding	13,834	0.07	4244	0.21	9590	0.01
Current breastfeeding	13,834	0.68	4244	0.89	9590	0.58
Milk or infant formula	14,815	0.51	4497	0.42	10,318	0.55
Health						
Low birthweight below 2.5 kg	15,142	0.06	4497	0.05	10,318	0.06
ANC with skilled personnel	15,142	0.76	4497	0.76	10,318	0.77
Childbirth with skilled personnel	15,142	0.88	4497	0.87	10,318	0.88
Birth by caesarean section	15,134	0.21	4497	0.21	10,315	0.21
Contraception	15,142	0.52	4497	0.46	10,318	0.55
Individual/household						
Male child	15,142	0.52	4497	0.51	10,318	0.52
Children ever born	15,142	3.27	4497	3.17	10,318	3.31
Dead children	15,142	0.15	4497	0.12	10,318	0.13
Primaparous mother	15,142	0.30	4497	0.33	10,318	0.29
Child marriage	15,142	0.35	4497	0.35	10,318	0.35
Husband-wife age difference	14,692	4.93	4372	4.94	10,023	4.92
Age of mother	15,142	27.80	4497	26.88	10,318	28.17
Primary education of mother	15,142	0.50	4497	0.50	10,318	0.50
Secondary education of mother	15,142	0.25	4497	0.23	10,318	0.26
Mother works	15,142	0.09	4497	0.08	10,318	0.09
Female household head	15,142	0.94	4497	0.94	10,318	0.95
Regional						
Urban	15,142	0.58	4497	0.57	10,318	0.58
Kurd	15,142	0.22	4497	0.21	10,318	0.22
Sunni	15,142	0.31	4497	0.33	10,318	0.30
Year is 2011	15,142	0.86	4497	0.84	10,318	0.87

### Breastfeeding status

Table 3 presents the probit regression estimates that constitute our main results. We have pooled the 2006 and 2011 observations to exploit all of the available variation in conflict levels. Our initial measure of conflict is the rate of casualties per 1000 persons in the governorate in which the household lives, averaged over the 3 years prior to survey administration. Robust standard errors, clustered at the level of the governorate, are given in parentheses. Marginal effects, evaluated at the mean, are given in brackets.

**Table 3:** Breastfeeding status

VARIABLES	Ever breastfed	Breastfed one hour after birth	Exclusively breastfeeding (< 6 months)	Currently breastfeeding (all ages)	Currently breastfeeding (< 6 months)	Currently breastfeeding (6–24 months)
Average casualty rate	− 0.306**	− 0.209	− 0.0973	− 0.205*	− 0.343**	− 0.167
	(0.126)	(0.293)	(0.306)	(0.l09)	(0.169)	(0.127)
	[−0.0375]	[− 0.0817]	[− 0.0274]	[− 0.0730]	[− 0.0609]	[− 0.0649]
Health						
Low birthweight (below 2.5 kg)	− 0.228***	− 0.0589	− 0.0687	− 0.186***	− 0.124	− 0.193***
	(0.0605)	(0.0491)	(0.113)	(0.0468)	(0.107)	(0.0608)
	[−0.0326]	[− 0.0230]	[− 0.0188]	[− 0.0686]	[− 0.0237]	[− 0.0763]
ANC with skilled personnel	0.163***	− 0.0939	− 0.128**	− 0.0340	0.0212	− 0.0304
	(0.0425)	(0.0660)	(0.0547)	(0.0321)	(0.0719)	(0.0267)
	[0.0214]	[− 0.0369]	[− 0.0370]	[− 0.120]	[0.00380]	[− 0.0118]
Childbirth with skilled personnel	− 0.0610	− 0.115	0.0390	− 0.0733*	− 0.0153	− 0.104**
	(0.0544)	(0.0798)	(0.0792)	(0.0392)	(0.109)	(0.0459)
	[−0.00722]	[−0.0454]	[0.0108]	[−0.0257]	[− 0.00270]	[− 0.0400]
C-section birth	− 0.256***	− 0.902***	− 0.181***	− 0.0597	− 0.174**	−0.0939**
	(0.0316)	(0.104)	(0.0640)	(0.0410)	(0.0772)	(0.0386)
	[−0.0353]	[− 0.315]	[− 0.0487]	[−0.0214]	[− 0.0329]	[− 0.0368]
Contraception	− 0.0475	0.00789	− 0.187***	0.0351	− 0.268***	0.207***
	(0.0302)	(0.0392)	(0.0528)	(0.0304)	(0.0535)	(0.0431)
	[−0.00581]	[0.00309]	[−0.0525]	[0.0125]	[−0.0482]	[0.0808]
Individual/household						
Male child	−0.0324	− 0.00191	0.0981***	0.0567**	0.0695**	0.0714**
	(0.0268)	(0.0191)	(0.0361)	(0.0236)	(0.0310)	(0.0316)
	[− 0.00398]	[−0.000750]	[0.0276]	[0.0202]	[0.0124]	[0.0278]
Children ever born	0.0607***	0.0124	0.0264	0.0596***	−0.00164	0.0511***
	(0.0100)	(0.00921)	(0.0180)	(0.0104)	(0.0395)	(0.0111)
	[0.00744]	[0.00487]	[0.00744]	[0.0212]	[−0.000292]	[0.0199]
Dead children	−0.447***	0.0294	0.00151	−0.114***	− 0.103	− 0.103***
	(0.0325)	(0.0236)	(0.0724)	(0.0319)	(0.0865)	(0.0390)
	[−0.0549]	[0.0115]	[0.000424]	[− 0.0407]	[− 0.0184]	[− 0.0403]
Primaparous mother	−0.0969*	− 0.109**	− 0.0966*	0.0692	− 0.0725	0.126***
	(0.0501)	(0.0447)	(0.0571)	(0.0485)	(0.115)	(0.0487)
	[−0.0122]	[− 0.0426]	[− 0.0268]	[0.0244]	[− 0.0131]	[0.0487]
Child marriage	−0.0897	0.00241	−0.112*	− 0.0336	0.00170	0.0294
	(0.0568)	(0.0287)	(0.0652)	(0.0272)	(0.0805)	(0.0335)
	[−0.0112]	[0.000945]	[−0.0311]	[− 0.0120]	[0.000302]	[0.0114]
Husband-wife age difference	0.00647	−4.71e-05	− 0.00569	− 0.00180	− 0.00264	− 0.00102
	(0.00421)	(0.00221)	(0.00363)	(0.00263)	(0.00692)	(0.00273)
	[0.000794]	[−1.84e-05]	[−0.00160]	[− 0.000640]	[− 0.000469]	[− 0.000398]
Age of mother	− 0.0144***	− 0.00117	− 0.0134***	− 0.00989***	0.00372	− 0.000320
	(0.00352)	(0.00289)	(0.00431)	(0.00299)	(0.00861)	(0.00404)
	[−0.00176]	[− 0.000457]	[− 0.00377]	[− 0.00352]	[0.000661]	[− 0.000125]
Primary education of mother	− 0.0285	− 0.107**	− 0.0464	−0.111***	− 0.0998	− 0.0903**
	(0.0404)	(0.0448)	(0.0782)	(0.0270)	(0.0714)	(0.0380)
	[−0.00350]	[− 0.0420]	[− 0.0131]	[− 0.0393]	[−0.0177]	[− 0.0352]
Secondary education of mother	−0.0682	− 0.0845	− 0.172***	− 0.241***	− 0.240***	− 0.228***
	(0.0471)	(0.0661)	(0.0652)	(0.0355)	(0.0814)	(0.0427)
	[−0.00756]	[− 0.0330]	[− 0.0464]	[− 0.0878]	[− 0.0462]	[− 0.0894]
Mother works	0.0291	0.0180	0.0209	4.71e-05	−0.0653	0.0148
	(0.0663)	(0.0883)	(0.0620)	(0.0489)	(0.113)	(0.0611)
	[0.00351]	[0.00707]	[0.00592]	[1.67e-05]	[−0.0120]	[0.00574]
Female household head	0.116	0.00323	0.239**	−0.128*	− 0.0631	− 0.139*
	(0.0702)	(0.0399)	(0.111)	(0.0669)	(0.128)	(0.0764)
	[0.0154]	[0.00127]	[0.0611]	[−0.0422]	[− 0.0108]	[− 0.0531]
Regional						
Urban	−0.186***	0.00626	−0.00783	− 0.0945**	− 0.130**	− 0.112**
	(0.0433)	(0.0634)	(0.0533)	(0.0385)	(0.0545)	(0.0456)
	[−0.0224]	[0.00245]	[−0.00220]	[− 0.0335]	[− 0.0230]	[− 0.0436]
Kurd	0.0307	− 0.359*	− 0.00890	− 0.407***	− 0.403***	− 0.471***
	(0.0820)	(0.184)	(0.136)	(0.0409)	(0.0470)	(0.0445)
	[0.00372]	[−0.136]	[− 0.00250]	[− 0.151]	[− 0.0825]	[− 0.186]
Sunni	−0.126	− 0.238	−0.119	− 0.102	−0.0565	− 0.165*
	(0.0956)	(0.212)	(0.156)	(0.0771)	(0.134)	(0.0897)
	[−0.0160]	[−0.0919]	[− 0.0329]	[− 0.0367]	[−0.0102]	[− 0.0648]
Year is 2011	−0.309	0.264*	−0.335***	− 0.257***	−0.186	− 0.267***
	(0.0827)	(0.139)	(0.0927)	(0.0852)	(0.117)	(0.0810)
	[−0.0319]	[0.101]	[−0.103]	[−0.0868]	[− 0.0306]	[− 0.101]
Constant	2.251***	0.219	−0.138	1.312***	1.930***	0.741***
	(0.125)	(0.205)	(0.239)	(0.0998)	(0.327)	(0.136)
Observations	14,701	13,562	4126	13,434	4126	9308

Robust standard errors in parentheses, clustered at level of governorate. Marginal effects in brackets, evaluated at the mean. *** *p* < 0.01, ** *p* < 0.05, * *p* < 0.1

For all indicators of breastfeeding status, conflict intensity is negatively associated with breastfeeding. Each 1-unit increase in the casualty rate (one additional casualty per 1000 persons) is associated with a 3.75 ppt decline in the probability that a child has ever been breastfed, an 8.17 ppt decline in the probability that a child was breastfed within 1 h after birth, a 2.74 ppt decline in the probability that an infant under 6 months of age is exclusively breastfed, and a 7.3 ppt decline in the probability that a child is currently breastfed. As for significance, the coefficient on the conflict variable is significant for the indicator reflecting whether a child was ever breastfed. The coefficient is borderline significant for the indicator reflecting current breastfeeding status among all children in the sample, but significant for the subsample of children who are less than 6 months of age. The conflict coefficients in the regressions for our other breastfeeding indicators are not significant at conventional levels.

**Fig. 4:**
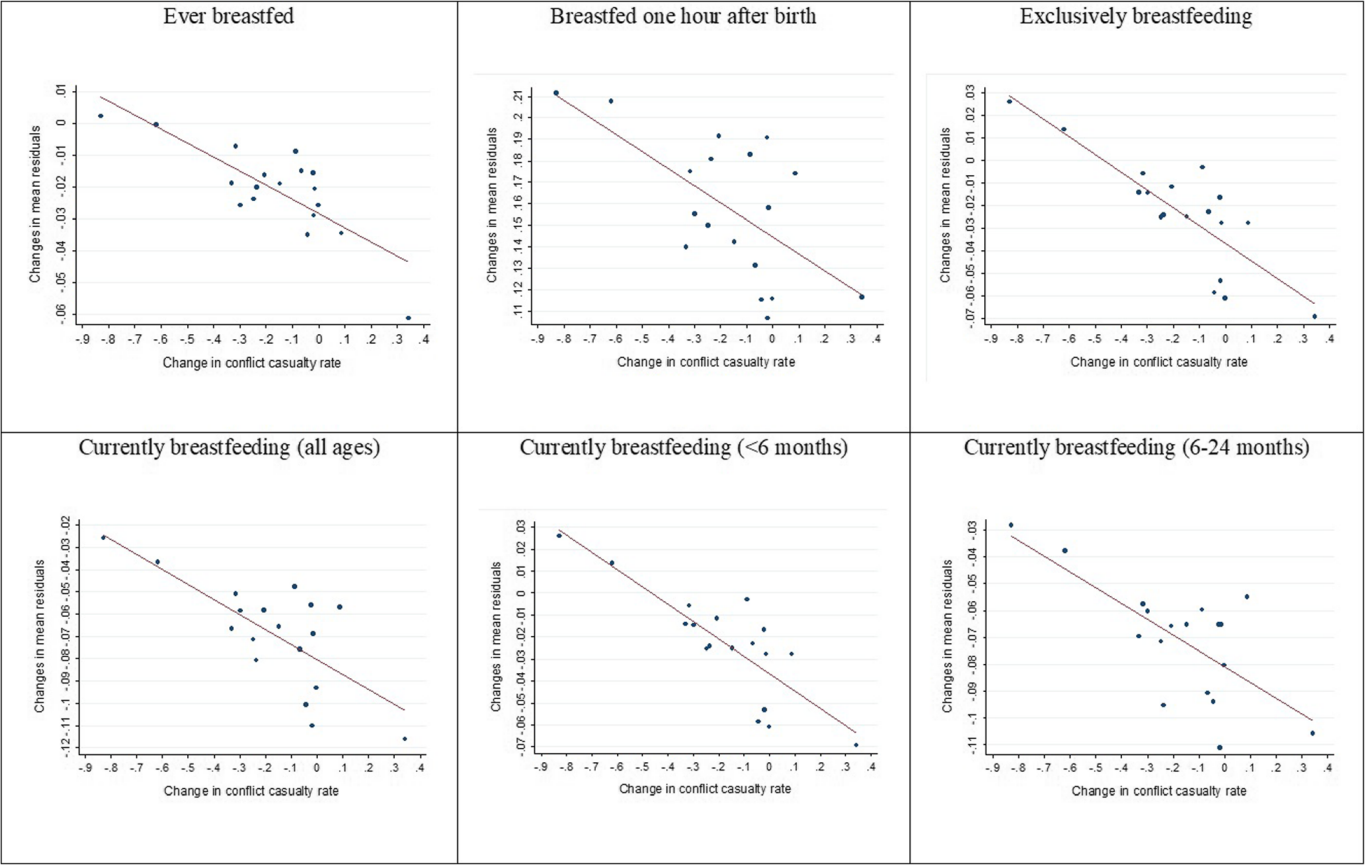
Regression residuals against changes in conflict, by governorate

As described in section 2.4, our first robustness check is to examine the relationship between *changes* in breastfeeding status and *changes* in conflict levels across our two survey years, at the level of the governorate. Figure 4 plots changes in the mean residuals from each of our six regressions, averaged over all observations in the governorate, against changes in the average casualty rate within the respective governorate. The main result is that the scatterplots display the expected downward slope, showing that governorates with larger increases in the casualty rate experienced larger declines in breastfeeding. Further, the pattern is distributed across most of the 18 governorates, and not driven by a few outliers.

Our second robustness check uses an alternative measure of conflict – the average rate of casualties in the 2 years prior to the child’s birth, rather than in the 3 years prior to survey administration. Table 4 presents the conflict coefficients from these regressions. The regressions employ the same controls as our main results, although we show only the coefficient on the conflict variable. For the pooled 2006 and 2011 sample, results are qualitatively similar to our main results. The casualty rate displays a negative association with all of our breastfeeding indicators. The coefficient is significant for the indicator reflecting whether a child was ever breastfed, as are the coefficients in the regressions of current breastfeeding status for all children and for the subsample of infants less than 6 months of age.

**Table 4:** Coefficient on conflict rate – Different measures of conflict, disaggregated by survey year

VARIABLES	Ever breastfed	Breastfed one hour after birth	Exclusively breastfeeding (< 6 months)	Currently breastfeeding (all ages)	Currently breastfeeding (< 6 months)	Currently breastfeeding (6–24 months)
Average casualty rate in 3 years prior to survey in governorate of household						
2006 and 2011 merged						
Casualty rate	−0.306**	− 0.209	− 0.0973	−0.205*	− 0.343**	−0.167
	(0.126)	(0.293)	(0.306)	(0.l09)	(0.169)	(0.127)
	[−0.0375]	[− 0.0817]	[− 0.0274]	[− 0.0730]	[− 0.0609]	[− 0.0649]
2011 only						
Casualty rate	− 0.430**	− 0.267	− 0.674	−0.416***	− 0.640***	−0.446***
	(0.197)	(0.486)	(0.582)	(0.0905)	(0.234)	(0.112)
	[− 0.0545]	[− 0.106]	[− 0.183]	[0.150]	[− 0.117]	[− 0.175]
2006 only						
Casualty rate	−0.201	− 0.186	0.144	0.00535	−0.287**	0.139
	(0.140)	(0.274)	(0.280)	(0.0761)	(0.137)	(0.0934)
	[−0.0184]	[−0.0625]	[0.0460]	[0.00168]	[−0.0445]	[0.0508]
Average casualty rate in 2 years prior to birth of child in governorate of household						
2006 and 2011 merged						
Casualty rate	−0.198**	− 0.127	− 0.223	−0.242**	− 0.0892	−0.405**
	(0.0995)	(0.160)	(0.103)	(0.110)	(0.0760)	(0.171)
	[−0.0225]	[− 0.0499]	[− 0.00626]	[− 0.0861]	[− 0.0159]	[− 0.158]
2011 only						
Casualty rate	−0.391	−0.558	− 0.785	−1.652***	−1.039	− 1.364***
	(0.325)	(0.551)	(1.232)	(0.364)	(0.648)	(0.260)
	[−0.0457]	[−0.221]	[− 0.213]	[− 0.595]	[−0.190]	[− 0.533]
2006 only						
Casualty rate	−0.200***	−0.0534	− 0.113	0.121	− 0.119	0.0835
	(0.0603)	(0.199)	(0.0894)	(0.101)	(0.0810)	(0.0814)
	[−0.0173]	[− 0.0180]	[− 0.0361]	[0.0378]	[− 0.0184]	[0.0304]

Robust standard errors in parentheses, clustered at level of governorate. Marginal effects in brackets, evaluated at the mean. *** *p* < 0.01, ** *p* < 0.05, * *p* < 0.1. All regressions use the same set of controls as in Table 3

Third, we disaggregate our analysis by year, and here we see some contrasts. These coefficients are also presented in Table 4. For the 2011 subsample, both of our conflict measures display a negative association with all of our breastfeeding indicators, which is consistent with our main results. Using our main conflict measure, these coefficients are significant for the indicator reflecting whether a child was ever breastfed and for the indicator reflecting current breastfeeding status, for children in both age groups. Using our alternative conflict measure, the conflict coefficient is not significant for the indicator reflecting whether a child was ever breastfed or for current breastfeeding status among infants less than 6 months old. For the 2006 subsample, using our primary measure of conflict, the coefficient is negative and significant for the indicator reflecting current breastfeeding status among infants less than 6 months old. By contrast, the estimated coefficient on conflict is positive (though insignificant) for the indicators reflecting exclusive breastfeeding, current breastfeeding status among all children in the sample and current breastfeeding status among children aged between 6 and 24 months. Using our alternative measure of conflict, the coefficient is negative and significant for the indicator reflecting whether a child was ever breastfed, but positive (though again insignificant) for the indicators reflecting current breastfeeding status among all children and current breastfeeding status among children aged between 6 and 24 months. We discuss these positive coefficient estimates, and the inconsistency in the 2006 and 2011 results, in section 4. However, note that all the positive coefficient estimates are from the 2006 subsample and that they are not significant.

Fourth, we add controls for household wealth. As discussed in section 2.4.4, a measure of household wealth is available only in the 2011 survey wave – an indicator by quintile. We present the results in Table 5. These regressions also include all of the controls from our main specifications. The results are similar to the main results that do not employ wealth controls. The conflict coefficient is negative and significant for the breastfeeding indicators reflecting whether a child was ever breastfed and whether a child is currently breastfed, for both age categories. This suggests that omitted variables bias from the absence of wealth controls in the main regressions is not a large concern. One other interesting aspect of these results is that breastfeeding is significantly more common among the households comprising the lowest quintile of the wealth distribution, a point to which we return in the discussion section.

**Table 5:** Breastfeeding status, including controls for household wealth (2011 only)

VARIABLES	Ever breastfed	Breastfed one hour after birth	Exclusively breastfeeding (< 6 months)	Currently breastfeeding (all ages)	Currently breastfeeding (< 6 months)	Currently breastfeeding (6–24 months)
Average casualty rate	−0.429**	− 0.260	− 0.663	− 0.420***	−0.515**	− 0.434***
	(0.206)	(0.501)	(0.495)	(0.0955)	(0.221)	(0.115)
	[−0.0544]	[−0.103]	[− 0.179]	[− 0.152]	[−0.0927]	[− 0.170]
Wealth						
Quintile 1	−0.0480	0.190*	0.0227	0.102*	0.121	0.137**
	(0.0837)	(0.0987)	(0.140)	(0.0539)	(0.107)	(0.0594)
	[−0.00615]	[0.0752]	[0.00616]	[0.0365]	[0.0213]	[0.0533]
Quintile 2	0.0177	0.0590	0.150	0.0847	0.109	0.0738
	(0.0863)	(0.0668)	(0.118)	(0.0532)	(0.109)	(0.0503)
	[0.00223]	[0.0234]	[0.0419]	[0.0302]	[0.0189]	[0.0288]
Quintile 3	0.00291	0.0325	0.0334	0.0105	0.00765	0.0237
	(0.0885)	(0.0858)	(0.119)	(0.0396)	(0.0800)	(0.0447)
	[0.000368]	[0.0129]	[0.00912]	[0.00377]	[0.00137]	[0.00926]
Quintile 4	0.0364	−0.0434	0.0356	−0.00290	0.0272	− 0.0213
	(0.0618)	(0.0426)	(0.122)	(0.0542)	(0.109)	(0.0469)
	[0.00453]	[−0.0171]	[0.00973]	[−0.00105]	[0.00483]	[−0.00835]
Other controls						
Health	Yes	Yes	Yes	Yes	Yes	Yes
Individual / Household	Yes	Yes	Yes	Yes	Yes	Yes
Regional	Yes	Yes	Yes	Yes	Yes	Yes
Constant	2.019***	0.276	−0.732***	0.999***	1.588***	0.394**
	(0.126)	(0.169)	(0.279)	(0.112)	(0.330)	(0.157)
Observations	12,653	11,646	3437	11,525	3437	8093

Robust standard errors in parentheses, clustered at level of governorate. Marginal effects in brackets, evaluated at the mean. *** *p* < 0.01, ** *p* < 0.05, * *p* < 0.1. All regressions use the same set of controls as in Table 3. Highest wealth quintile omitted

Finally, we directly study the provision of breastmilk substitutes feeding by defining a dependent variable equal to 1 if a child consumed milk or infant formula in the day preceding the survey. We restrict the sample to infants under 6 months old, to focus on the period for which exclusive breastfeeding is recommended, and to the 2011 survey wave, for which household wealth information is available. The probit regression uses all the controls included in our main regressions. The results are given in Table 6, and they indicate that an increase in armed conflict is associated with an increase in milk and formula provision, although the coefficient is not significant at conventional levels. Interacting casualty rates with wealth quintiles, however, shows differentiation by household wealth, with conflict levels more strongly associated with increased use of BMS among wealthier households.

**Table 6:** Provision of milk or infant formula, children less than 6 months old

	Dependent variable: Child given milk or infant formula				
VARIABLES	(1)	(2)	(3)	(4)	(5)
Average casualty rate	0.394	0.288	0.294	0.156	0.245
	(0.367)	(0.298)	(0.271)	(0.280)	(0.507)
	[0.155]	[0.114]	[0.116]	[0.0616]	[0.0967]
Casualty*Quintile 1	−0.285				−0.147
	(0.355)				(0.521)
	[−0.112]				[−0.0579]
Casualty*Quintile 2		−0.0558			−0.0193
		(0.243)			(0.338)
		[−0.0220]			[−0.00760]
Casualty*Quintile 3			−0.135		−0.0830
			(0.293)		(0.425)
			[−0.0530]		[− 0.0327]
Casualty*Quintile 4				0.850**	0.764
				(0.380)	(0.493)
				[0.335**]	[0.301]
Other Controls					
Health	Yes	Yes	Yes	Yes	Yes
Individual/household	Yes	Yes	Yes	Yes	Yes
Regional	Yes	Yes	Yes	Yes	Yes
Constant	−0.497**	−0.466**	−0.463**	−0.442**	−0.463*
	(0.208)	(0.206)	(0.205)	(0.204)	(0.248)
Observations	2945	2945	2945	2945	2945

Robust standard errors in parentheses, clustered at level of governorate. Marginal effects in brackets, evaluated at the mean. *** *p* < 0.01, ** *p* < 0.05, * *p* < 0.1. All regressions use the same set of controls as in Table 3

### Breastfeeding duration

We turn now to a complementary analysis of breastfeeding duration, using a Cox proportional hazards model. The results are given in Table 7. For the pooled sample and for the 2011 subsample, an increase in conflict-related casualties is associated with a higher hazard rate of breastfeeding termination, and thus shorter breastfeeding duration. The coefficient is significant for the 2011 subsample. The calculated hazard ratio for the full sample is 1.174, reflecting a 17.4 ppt increase in the risk of breastfeeding termination associated with a unit increase in the average casualty rate (similar to moving from the safest area to the most dangerous area). That is, a mother living in a conflict-prone area is 17.4 ppts more likely to stop breastfeeding in any given month than a mother living in an area with no conflict-related casualties. However, it is important to note that the directional result is different across years. For the 2006 subsample, higher conflict intensity is associated with longer breastfeeding duration, and the coefficient is significant. This result is consistent with some of the positive (though insignificant) coefficients obtained for the breastfeeding status regressions run on the 2006 subsample.

**Table 7:** Breastfeeding duration

	All years		Year 2006		Year 2011	
Variables	Coefficient	Hazard ratio	Coefficient	Hazard ratio	Coefficient	Hazard ratio
Average casualty rate	0.119	1.1737	−0.270***	0.7633	0.463***	1.7221
	(0.175)		(0.0949)		(0.142)	
Health						
Low birthweight (below 2.5 kg)	0.137**	1.1613	0.0861	1.0900	0.144**	1.1785
	(0.0557)		(0.141)		(0.0583)	
ANC with skilled personnel	0.0745**	1.0918	0.141	1.1509	0.0747**	1.0929
	(0.0333)		(0.228)		(0.0361)	
Childbirth with skilled personnel	0.146***	1.1804	0.0859	1.0897	0.163***	1.2044
	(0.0397)		(0.210)		(0.0397)	
C-section birth	0.119**	1.1178	−0.0558	0.9457	0.140**	1.1403
	(0.0532)		(0.0930)		(0.0614)	
Contraception	−0.105***	0.9146	−0.327***	0.7214	−0.0916***	0.9166
	(0.0335)		(0.114)		(0.0334)	
Individual/household						
Male child	−0.0996***	0.9125	−0.118*	0.8888	−0.0971***	0.9160
	(0.0270)		(0.0611)		(0.0298)	
Children ever born	0.0273*	1.0312	0.644***	1.9049	0.0341***	1.0391
	(0.0145)		(0.172)		(0.0118)	
Dead children	0.0177	1.0162	−0.706**	0.4934	0.0235	1.0219
	(0.0326)		(0.294)		(0.0340)	
Primaparous mother	0.0417	1.0344	0.272	1.3132	0.0431	1.0355
	(0.0572)		(0.193)		(0.0608)	
Child marriage	−0.224***	0.7999	−0.223	0.8001	−0.219***	0.8035
	(0.0380)		(0.161)		(0.0369)	
Husband-wife age difference	−0.00383	0.9961	0.00264	1.0026	−0.00488*	0.9949
	(0.00239)		(0.0108)		(0.00277)	
Age of mother	−0.0371***	0.9623	−0.0151	0.9850	−0.0414***	0.9578
	(0.00483)		(0.0158)		(0.00434)	
Primary education of mother	0.0565	1.0678	−0.134	1.8746	0.0799**	1.0948
	(0.0345)		(0.102)		(0.0353)	
Secondary education of mother	0.171***	1.1876	−0.0867	0.9070	0.204***	1.2283
	(0.0480)		(0.112)		(0.0552)	
Mother works	0.0254	1.0352	−0.130	0.8780	0.0216	1.0305
	(0.0746)		(0.109)		(0.0772)	
Female household head	0.00116	1.0242	−0.149	0.8615	0.0460	1.0813
	(0.0929)		(0.151)		(0.113)	
Regional						
Urban	0.0450	1.0537	0.133	1.1420	0.0406	1.0497
	(0.0444)		(0.107)		(0.0460)	
Kurd	0.405***	1.5240	0.383***	1.4668	0.410***	1.5363
	(0.0442)		(0.128)		(0.0491)	
Sunni	0.216**	1.2249	0.173	1.1888	0.118	1.0976
	(0.108)		(0.180)		(0.113)	
Year is 2011	0.0240	1.0233				
	(0.0177)					
Observations	13,812		1924		11,888	

Robust standard errors in parentheses, clustered at level of governorate. *** *p* < 0.01, ** *p* < 0.05, * *p* < 0.1

### Control variables

Referring back to our main results in Table 3 and in Table 7, increases in education levels are associated with a decline in the prevalence and duration of breastfeeding, as is residence in an urban area. Mothers who have had more children are more likely to breastfeed, while mothers who have experienced the death of a child are less likely to breastfeed. Mothers are more likely to breastfeed male children, and to breastfeed them for a longer period. Finally, mothers in governorates that are predominantly Kurdish or Sunni are less likely to breastfeed than mothers in Shia areas, and when they do the duration is shorter.

## Discussion

### Interpretation of results

The main results show a statistically significant negative association between conflict-related casualties and an indicator reflecting whether a child was ever breastfed. There is also a negative association with an indicator reflecting whether a child is currently breastfed, borderline significant for all children in the sample, but significant for infants under the age of 6 months. One implication, combining these findings, is that at least part of the association between breastfeeding status and conflict is attributable to breastfeeding never being initiated at all, rather than to early termination.

To interpret the economic significance of our main results, a good indicator of magnitude is to evaluate the estimated breastfeeding probabilities at the lowest and at the highest conflict levels in our sample, and to compare the two. For our sample, the lowest rate of conflict-related casualties was almost zero in both 2006 and 2011, while the most dangerous governorate saw an annual average of 1.13 conflict-related casualties per 1000 persons, over the 3 years prior to the survey. This level of increase in casualties is associated with an estimated 8.5 ppt decline in the probability that a child is currently breastfed and an estimated 5.04 ppt decline in the probability that a child has ever been breastfed. This exercise allows us to compare the magnitude of our results with other results obtained in the literature, which typically involve a discrete contrast between conflict areas and non-conflict areas. In fact, our results are not far from those obtained by Guerrero-Serdan [32], who estimated a 7 ppt decline in breastfeeding associated with residing in a conflict area, or by Zakanj et al. [66], who obtained an estimate of 4.2 ppt based on comparing war-free and war-affected areas in Croatia. We can also compare these with estimates of the impact of war on other household variables in Iraq. The impact of conflict on breastfeeding is apparently large compared to the impact on female education. UNICEF [58] finds that female primary school enrollment had dropped in all areas of Iraq by 2007 and, while the decline was sharper in conflict-affected areas, the largest decline was only 0.92 ppt. On the other hand, Cetorelli [15] finds that children in war-affected areas are 21.5 ppts less likely to be vaccinated against polio than children in safer areas.

While our results do reveal some potentially illuminating features of the relationship between conflict and breastfeeding, we should note that they are not consistently significant or unidirectional. Initiation of breastfeeding within 1 h and exclusive breastfeeding are not significantly associated with conflict rates in our main results or in any of the variations on our main results presented in the robustness checks. Furthermore, as noted in section 3.2, some estimated coefficients are positive. However, the positive coefficient estimates are all from the 2006 subsample and none is significant. Generally, the 2011 results are more robust than the 2006 results. One potential explanation is that the sample size from 2011 is more than six times larger. A second potential explanation is that 2006 was the apex of the conflict. Measuring casualties accurately was likely more challenging then, and presumably communications networks improved over the intervening years. Also, positive coefficients are not theoretically implausible, an issue to which we return in section 4.2.

Other results are generally consistent with the literature. While the association between education and breastfeeding is positive in developed countries, we find a negative correlation, and other authors have noted this same result for poor countries [16]. This link is typically attributed to a rejection of traditional modes of child care among educated women [1]. Other authors have also documented a negative association between breastfeeding and a mother’s age and her residence in an urban area, attributed variously to less reliance on extended family among older and city-dwelling mothers [1] and to marketing of infant formula in urban areas [51]. Educated and urban mothers are also more likely to work, which would increase their opportunity cost to breastfeed. Regarding parity, Dennis [19] previously found that prior breastfeeding success is a strong predictor of duration of future breastfeeding, consistent with our finding that women who have had more children are more likely to breastfeed. Our finding that mothers who have experienced the death of a child are less likely to breastfeed is a logical inversion of Dennis’ premise. The bias towards breastfeeding boys, and for longer periods, could reflect heavier investment in male children, which is well-known in Arab societies [49]. Finally, regarding sect, our result that mothers in predominantly Shia governorates are more likely to breastfeed, and for longer, than mothers in Kurdish and Sunni governorates, is instructive in the context of Iraq. Shia in Iraq are known to have more traditional Islamic values than their Kurdish and Sunni counterparts. For example, they more likely to support the merging of religion and state, whereas Kurds and Sunnis support secular government [55]. In Lebanon, which also features large populations of both Muslim sects, Faour [27] finds higher fertility and lower use of contracteption among the Shia, and Mazrui [42] finds the same with respect to African Muslims.^4^ Meyer et al. [43] find in Kuwait that Shia religious institutions are more likely than Sunni institutions to promulgate an interpretation of Islam that is hostile to political inclusivity for women. While we were not able to find any literature that directly addresses breastfeeding practices by sect in Islam, Huffman et al. [36] find shorter breastfeeding duration among Muslims than among Hindus in Bangladesh. In any case, if Shia are more religious than Sunnis on average, then conformity with religiously-prescribed breastfeeding practices could help to account for higher rates of breastfeeding in Shia-dominant areas.

### Transmission mechanisms

In this section, we discuss the various channels through which armed conflict might alter the prevalence and duration of breastfeeding. First, stress is known to reduce the quantity and quality of breastmilk production. Grajeda and Perez-Escamilla [31] find that stress, measured objectively via Cortisol levels, is associated with delayed onset of lactation and subsequently with shorter breastfeeding duration. Keith et al. [38] provide evidence that interventions to reduce stress increase both the production and the fat content of breastmilk. It is also possible that conflict-affected mothers may lack a suitably private and comfortable location to breastfeed or pump, especially if displaced, further reducing breastfeeding frequency and milk production. Overall, mothers affected by conflict-related stress may see the production and quality of their breastmilk as inadequate. Even if this is not the case in reality, many mothers believe that their breastmilk is “insufficient to satisfy their infant’s needs” [46]. In other words, regardless of whether Insufficient Milk Syndrome (IMS) is real or imagined, many mothers may opt to not breastfeed their children, or to terminate breastfeeding earlier than recommended. In addition, conflict generates a large number of refugees and displaced individuals who are likely to face difficulties in accessing basic healthcare and day-to-day necessities [17].^5^ This could further impair a mother’s nutritional intake and the quality of her breastmilk, leading to a decline in breastfeeding.

Conflict can also reconfigure family dynamics, which impacts breastfeeding. Male war-related casualties endow women with more responsibilities inside the household and increased need for income-generating activities outside the household, which in turn reduces their time or inclination to breastfeed [26]. Mothers in this situation may also be compelled to spend significant portions of the day acquiring necessities like food and fuel. Reductions in household income for these female-headed households could be another channel through which food and nutritional intake is curtailed, leading to a decline in breastfeeding. Such situations are especially pronounced in times of conflict, where mothers are often the first in a family to sacrifice their own food intake if faced with a shortage, so as to provide for other family members [26].

Supply-side effects in health care provision constitute another potential transmission mechanism. Looting and destruction, inadequate supplies, a shortage of female medical staff and insufficiently trained personnel were common in the healthcare sector following the US invasion [44]. Even a decade subsequent to the invasion, the displacement of middle-class Iraqis has presumably reduced the supply of health-care personnel and thus the ability of mothers to access quality care. Furthermore, lack of medical support creates severe gaps in the transfer of knowledge to mothers about the importance of breastfeeding [26]. Indeed, the results in Del Bono and Rabe [18] and Fitzsimons and Vera-Hernandez [29] point to such support from health professionals as a key determinant of breastfeeding. Surrounded by untrained personnel and personnel in insufficient numbers generally, a rift in knowledge created by violent conflict can help to account for reduced prevalence of breastfeeding. The effect is likely to be even stronger if the mother is displaced herself.

Maternal health presents another channel from violent conflict to breastfeeding, as mothers who are unwell are less likely to breastfeed. We do not have sufficient information on maternal health to test this hypothesis directly, but it is worth noting in Table 3 that low birthweight of the baby has a negative and significant association with subsequent breastfeeding, which is suggestive about differences in mothers’ health.^6^ Many pregnant women in Iraq are anemic [64]. Moreover, the maternal mortality ratio in Iraq is substantially higher than in many regional counterparts [65]. Even if the mother does breastfeed, poor nutritional status owing to disrupted supply chains can reduce the volume, as well as the fat and vitamin content of her milk [47].

Finally, we explore the role of breastmilk substitutes such as formula as an obvious intermediary in the relationship between conflict and breastfeeding practices. Our results show that breastfeeding is most common among the poorest households and among mothers with lower levels of education, perhaps reflecting less disposable income to purchase BMS. Breast milk contains various antibodies, enzymes and hormones that are absent in infant formula [39], so substitution of formula for breastmilk is potentially harmful. Additionally, Guerrero-Serdan [32] documents a serious deterioration in Iraq’s sanitation and clean water infrastructure, introducing another potential source of risk from formula-feeding, owing to the need to use water to reconstitute infant formula.

The institutional context in Iraq is worth mentioning. The World Food Programme included significant quantities of infant formula in its food distributions in Iraq, beginning in 1996, for all infants in the first year of their lives. Few instructions on bottle-feeding were provided, and the formula was often reconstituted with dirty water. A 2003 report by the Emergency Nutrition Network determined that these formula distributions were “a major contributing factor to infant malnutrition, morbidity and mortality”, and that they reduced breastfeeding. Teshome and Takea [56] note that routine formula distribution only ended in 2017 after intense lobbying motivated in part by concern about low breastfeeding rates. They also cite an ongoing lack of support resources, especially trained staff, to promote optimal breastfeeding among mothers.

Overall, conflict-related circumstances can lead to a lower probability and shorter duration of breastfeeding, for the reasons discussed above. Richer households have a straightforward solution available, because breastmilk substitutes are more affordable and accessible to them. Thus, it may actually be that infants from poor households suffer less in this regard, since substitution to BMS is less of a viable option for these families,^7^ especially if the level of violence is so severe that it compromises the distribution of aid by humanitarian organizations. At the very least, our results suggest that policies to encourage breastfeeding in conflict areas should consider varying breastfeeding patterns and determinants per conflict intensity, household wealth, and the age of the child. This analysis may also offer some insight into the positive (though insignificant) coefficient estimates from the 2006 sample, which was the peak of the conflict. That is, conflict intensity may be generally associated with reduced breastfeeding, but the effect is most pronounced when breastmilk substitutes are easily available. If conflict is so severe that substitutes like formula are difficult to acquire then the negative effect on breastfeeding might be dampened, or even reversed, out of a lack of any other viable feeding options.^8^ Our analysis suggests that this seemingly counterintuitive positive association is most likely among families with limited economic means who reside in areas with the most serious conflict. Of course, it is also possible that serious conflict leads to a decline both in breastfeeding and in the availability of BMS, leading to malnutrition among affected infants. Haidar et al. [33] document high incidence of infant malnutrition in some war-affected areas of Iraq and counsel wider distribution of formula to alleviate the problem. Ververs et al. [62], in response, reiterate the need for caution in the use of formula provision as a solution.

### Limitations

A potential limitation of this study is that our measure of conflict intensity is drawn from the IBC database, which documents war-related casualties. IBC figures are likely to understate the true extent of armed conflict due to its focus on media sources for much of its information and the inherent selection bias in this process [50, 63]. Nevertheless, the dataset offers insight into the magnitude and geographical attributes of casualties in the Iraq conflict, and remains one of the most reliable sources on the matter [14, 28]. It would also be useful to have more localized measures of conflict.

A second potential limitation is the confounding effect of migration. As we noted in section 2, the random sampling in MICS records individuals, including displaced persons, at their current locations. A mother who is displaced from a high-conflict area to an area with a lower level of conflict may still be suffering from the effects of conflict at her original home. While our dataset does not allow us to explore this possibility directly, it would tend to create an *upward* bias in the coefficient of interest since such mothers are affected by conflict, but not living in conflict zones at the time of the survey. That is, if this is a serious issue in our dataset, it works against our main result and suggests that the association between conflict and breastfeeding is even stronger. Our dataset does reveal some information along these lines – The 2011 sample is more heavily concentrated in urban areas and in Sunni and Kurdish governorates than is the 2006 sample. However, there is no micro-level data on displacement at the family level.

A third potential limitation is missing information in the MICS survey that would be potentially useful in addressing additional confounding effects. Very limited information on household wealth is provided in the 2011 survey, and none in the 2006 survey. Furthermore, religious divides are a complicated issue in Iraq, and the dataset does not record any direct information about religion or sect. Instead, we rely on the majority sect in the governorate in which the respondent resides as a proxy.

Fourth, reverse causation is always a potential confounding factor. The relationship between reductions in breastfeeding and increases in the use of BMS is complex, and the child’s health status is also a confounding factor. A mother with a sick child might be more likely to rely on BMS. Unfortunately, the dataset lacks detailed information on treatment for other child health conditions that would be good indicators of child morbidity.

Finally, there is a possibility of endogeneity owing to omitted variables bias. Households in different governorates may possess unobserved attributes that are correlated with conflict levels and that also impact breastfeeding practices. We did employ an instrumental variables analysis, using distance from border crossings with Syria and Iran (hotspots for weapons smuggling and terrorism) as instruments for casualty rates in the governorate, and results were similar to our main results.^9^ Conflict levels display a negative and significant association with the probability that a child was ever breastfed, and a marginally significant negative association with the probability that an infant under the age of 6 months is currently breastfeeding.^10^ Another approach to addressing issues of endogeneity would be repeated sampling of the same households in future survey waves, allowing researchers to implement panel analysis.

### Conclusions

Around seven million children under the age of 5 die each year around the world. Almost half of these are newborns, and scores of these deaths are estimated to have been preventable through the simple act of breastfeeding [59]. While typical determinants of breastfeeding include individual and household-level characteristics, this study goes a step further to consider exposure to war. Iraq, and the Middle East in general, have experienced high levels of violent conflict in the last several decades and this kind of tension is unfortunately becoming part of the natural landscape in the region. Using household data from Iraq, we analyzed the relationship between breastfeeding and the level of violent conflict. While there are some mixed results, our findings generally indicate a negative association between exposure to armed conflict and breastfeeding status. There is no doubt that conflict creates immediate and dire devastation to human life, infrastructure and the economy. But conflict can also shape the health of future generations and lead to longer-term social and economic consequences. In this paper, we have argued that war can alter breastfeeding practices for women in conflict zones, which represents an important and almost completely unexplored dimension of these longer-term consequences.

Moreover, our paper explores transmission mechanisms that highlight differences in breastfeeding status based not just on conflict intensity, but also on its interaction with household wealth and the provision of breastmilk substitutes. Our results suggest an increase in provision of BMS by women in wealthier quintiles concurrent to high levels of conflict. This finding conversely implies a potential ameliorative effect for poorer households through a lower reliance on BMS and expanded utilization of breastfeeding.

Our study’s evidence regarding the relationship between conflict and infant feeding, and consequently on the early health of children, is an important path of inquiry for policymakers and especially for international organizations that support interventions aimed at advancing maternal and child health in conflict zones. Formula distribution creates the impression of offering temporary relief for trapped families, but it introduces new risks; reliance on dirty water to reconstitute formula and reducing the probability and duration of breastfeeding can harm child health. Thus, alternative interventions like skilled breastfeeding support for conflict-affected mothers may provide more sustainable benefits and with fewer unintended consequences. However, we want to emphasize that for infants who *are* dependent on formula during and after conflict periods, it is important to provide appropriate information and support in complement to formula distributions. Such support requires investment in the supply of healthcare and targeted support for infants and mothers. Interventions aimed at promoting breastfeeding in conflict areas are especially challenging in contexts where rates of breastfeeding are already low, and they require a sustained commitment by aid organizations [25]. This is consistent with earlier literature that counsels interventions stressing the importance and benefits of breastfeeding for child health and for the future health of the population; we emphasize here that the need is especially profound during conflict periods. Attention to the supply of health care and to support systems for women, including skilled breastfeeding support, are matters of the utmost urgency both during and after conflict periods.

## Data Availability

The datasets used and/or analysed during the current study are available from the corresponding author on reasonable request.

## Abbreviations

IBC: Iraq Body Count
IMS: Insufficient milk syndrome
MENA: Middle East and North Africa
MICS: Multiple indicator cluster surveys
UNICEF: United Nations International Children’s Emergency Fund
US: United States (of America)
WHO: World Health Organization

## Footnotes

1 The MENA region has experienced the largest number of conflicts in any major region worldwide since World War II [45], with at least 183,535 civilian casualties in Iraq directly related to the conflict that began there in 2003 (Iraq Body Count).

2 Contraception is a useful control as a proxy for preferences related to childbearing. However, one concern is that the definition of contraception does include the lactational amenorrhea method (LAM), which induces a direct impact on breastfeeding practices. We did try dropping this variable on account of the potential misspecification, and the results were almost the same.

3 We include the age difference between the husband and the wife as a proxy for traditional values or power inequalities within the family, which may be correlated with time allocation, and with breastfeeding choices specifically.

4 This result is not universal, however. Shia in Iran have a lower fertility rate (Pew 2011).

5 Generally, conflict can reconfigure the way that people interact with the health care system in a variety of ways [20].

6 Probit regressions on our sample also show a negative and significant association between a mother’s age and giving birth to a low-birthweight baby, i.e. babies with low birthweight (< 2.5 kg) are more common among younger mothers.

7 However, these effects appear not to continue past 6 months of age. Probit regressions on our sample run on the older cohort (6–24 months) find that, for children in poorer households, intense conflict is associated with both reduced probability of breastfeeding and with reduced consumption of milk and infant formula

8 Some civilian areas were under a literal blockade at the height of the conflict.

9 This instrument is common in the literature. See, for example, Akresh and De Walque [3], Arcand and Wouabe [9], Serneels and Verporten [53] and Diwakar [23].

10 F-statistics from the first-stage OLS regressions are well outside of the range that would suggest a problem with weak instruments.
